# Serial daily lactate levels association with 30-day outcome in cardiogenic shock patients treated with VA-ECMO: a post-hoc analysis of the HYPO-ECMO study

**DOI:** 10.1186/s13613-024-01266-6

**Published:** 2024-03-27

**Authors:** Bruno Levy, Nicolas Girerd, Guillaume Baudry, Kevin Duarte, Samuel Cuau, Jan Bakker, Antoine Kimmoun, for the HYPO-ECMO trial group and the International ECMO Network (ECMONet)

**Affiliations:** 1 Université de Lorraine, CHRU Nancy, Médecine Intensive et Réanimation, Nancy, France; 2 Université de Lorraine, INSERM U1116, Nancy, France; 3 Université de Lorraine, CIC-P INSERM 1433, INI-CRCT F-CRIN Network, Nancy, France; 4 NYU Langone and Columbia University Medical Center, New York, USA; 5 Erasmus MC University Medical Center Rotterdam, Rotterdam, Netherlands; 6 Pontificia Universidad Católica de Chile, Santiago, Chile

**Keywords:** Cardiogenic shock, Lactate, Extracorporeal Membrane Oxygenation, Hypothermia

## Abstract

**Background:**

Reliable predictors of outcomes in venoarterial extracorporeal membrane oxygenation (VA-ECMO) therapy are limited. While elevated lactate levels over time have been linked to outcomes in cardiogenic shock (CS), their significance in VA-ECMO-treated patients remains inconclusive.

**Methods:**

We conducted a post hoc analysis of data from the HYPO-ECMO trial, which compared normothermia to moderate hypothermia in CS patients supported by VA-ECMO. We examined daily lactate levels collected over a week to assess their correlation with 30-day mortality.

**Results:**

Among the 318 out of 334 patients (95%) with baseline lactate measurements, 66 had normal levels (< 2.2 mmol/l, 21%). No difference was found in lactate course between moderate hypothermia and normothermia groups. Lactate levels were consistently higher in non-survivors at each time point (p = 0.0002). Baseline hyperlactatemia was associated with an increased risk of death (Hazard Ratio [HR]: 1.85 (1.12–3.05), p = 0.016). When considering all time points, lactate levels during the ICU stay were significantly and gradually associated with a higher risk of death (p < 0.0001). In the overall population, a decrease in lactate levels was not linked to 30-day mortality. However, patients with baseline hyperlactatemia exhibited a more significant decrease in lactate levels from day one to seven (p < 0.0001). In this group, survivors had a significantly greater decrease in lactate levels at day 1 compared to non-survivors (63% (48–77) versus 57% (21–75), p = 0.026). Patients experiencing a secondary increase in lactate (24%) had a worse prognosis (Hazard Ratio: 1.78 (1.21–2.61), p = 0.004), regardless of both baseline lactate levels and the occurrence of severe ischemic adverse events (intestinal and/or limb ischemia).

**Conclusions:**

The consistent and significant association between lactate levels, whether assessed at baseline or during ICU treatment, and the risk of mortality underscores the pivotal prognostic relevance of lactate levels in patients with CS undergoing VA-ECMO therapy. The study findings provide some novel insights, regarding the trend profile and the relevance of a second peak during the 7 day period after ECMO start.

*Trial Registration* identifier NCT02754193 registered on 2016–04–12.

**Supplementary Information:**

The online version contains supplementary material available at 10.1186/s13613-024-01266-6.

## Background

The interpretation of lactate levels and their changes over time in circulatory shock is intricate, involving assessments of both production and clearance. Lactate in shock primarily originates from two processes: pure anaerobic metabolism, which is rare except in cases of cardiac arrest or severely reduced blood flow and exaggerated aerobic glycolysis [1, 2]. The latter is driven by inflammation and epinephrine effects through β-2 adrenoreceptors, along with the activation of the Na–K + ATPase pump, making it theoretically independent of oxygen deficiency [3]. Lactate metabolism is a multifaceted process involving the liver and kidney functions and various pathways. Like glucose, lactate can also serve as a metabolic substrate. Particularly during stress, such as sepsis, lactate, through several "shuttles," becomes a significant cellular energy source [4]. Despite these complexities, elevated lactate levels, often referred to as lactate clearance or time to lactate normalization, have shown associations with prognosis in various scenarios, including septic shock, hemorrhagic shock, and cardiogenic shock (CS) [5–9]. The prognostic significance of lactate level in non-assisted CS is well established [10]. CS patients supported with venoarterial extracorporeal membrane oxygenation (VA-ECMO) present unique challenges that might complicate lactate kinetic interpretation. First and compared to medical treatment only, during VA-ECMO, peripheral blood flow is rapidly and efficiently restored. They commonly experience substantial ischemic-reperfusion injuries, receive high doses of catecholamines, and frequently suffer from both liver and kidney dysfunction based on the severity and duration of low cardiac output syndrome. Additionally, approximately 50% of these patients have undergone cardiac arrest [11]. Furthermore, the degree and reversibility of myocardial failure and complications associated with VA-ECMO may influence the relationship between lactate levels, changes in lactate, and time to lactate normalization, and early or late survival. Pre-VA-ECMO lactate levels have been linked to prognosis [12, 13] or included in several prognostic scores [14, 15], but data regarding the evolution of lactate or its derivatives during VA-ECMO treatment are scarce [16]. Therefore, utilizing data from the recently published HYPO-ECMO trial (Effect of Moderate Hypothermia vs. Normothermia on 30-Day Mortality in Patients With Cardiogenic Shock Receiving Venoarterial Extracorporeal Membrane Oxygenation: A Randomized Clinical Trial), we aimed to investigate whether baseline lactate levels, changes in lactate levels, and the reduction of lactate levels are associated with survival in patients with CS treated with VA-ECMO. This information could potentially serve as a reasonable therapeutic target for clinicians and as surrogate endpoints for researchers.

## Methods

### Study design

The design and primary results of the HYPO-ECMO study have been reported previously [11, 17]. Briefly, the objective of HYPO-ECMO was to determine whether early use of moderate hypothermia (33–34 °C) compared with strict normothermia (36–37 °C) improved mortality in patients with CS receiving VA-ECMO. All patients treated with VA-ECMO were screened for enrollment. Patients were eligible for enrollment if they were endotracheally intubated and had been receiving VA-ECMO for less than six hours.

Exclusion criteria were age < 18 years, pregnancy, VA-ECMO after cardiac surgery for heart or lung-heart transplantation or left ventricular assist device implantation, VA-ECMO for acute poisoning, uncontrolled bleeding (bleeding despite medical intervention, surgery or medication), VA-ECMO in the context of cardiopulmonary resuscitation for > 45 min, out-of-hospital refractory cardiac arrest, participation in other interventional research involving therapeutic interventions, patients under guardianship, clinician-based assessment of irreversible neurologic injury, and a decision to withhold or withdraw life-sustaining therapies.

The trial was supported by the international ECMO Network (ECMONet) and was approved by French health authorities (Agence Nationale de la Sécurité du Médicament et des Produits de Santé) and the appropriate ethics committee (Comité de Protection des Personnes). In accordance with French law, the ethics committee waived the requirement for informed consent prior to inclusion from patients because they were being treated in an emergency setting and were unable to provide informed consent.

### Hypo-emo study endpoints

The primary outcome was mortality at day 30 after inclusion. Main secondary outcomes were (1) mortality at days 7, 60, and 180, (2) a composite outcome of death, heart transplant, escalation to left ventricular assist device implantation, or stroke at days 30, 60, and 180 (3) Use of renal replacement therapy.

### Post-hoc study outcomes

The primary goal of this post hoc analysis was to determine the association of lactate levels measured over the first seven days with 30-days survival in patients with CS supported with VA-ECMO.

### Definition and timepoints

Hyperlactatemia was defined as a lactate level > 2 mmol/L. Normolactatemia was defined as a lactate level ≤ 2.2 mmol/L. Lactate levels were measured at baseline (just before ECMO implantation) and the following seven days (morning labs at 8 AM) as reported previously [11]. A late lactate increase was defined as a lactate increase preceded by at least one normal lactate value. Lactate clearance is the removal of lactate from blood, expressed as a volume (milliliters) over time (minutes). The so-called lactate clearance in the ICU literature is defined as the initial lactate minus subsequent lactate/initial lactate × 100%. The use of this former definition is confusing, as lactate clearance should be defined as the metabolism of lactate [7]. Therefore, it is recommended to use the term decrease in lactate level, since it reflects the end result of both lactate production and consumption [18]. Therefore, the decrease in lactate level (%) was defined by the following formulae: $[\left( {{\text{lactate at time point of interest}}--{\text{initial lactate}}} \right)/{\text{initial lactate}}*{1}00$.

Ischemic adverse events were prospectively recorded and checked by the clinical trial oversight.

### Statistical analysis

#### Data description

Analytical data are presented as medians with 25th and 75th percentiles (median (interquartile range)) for continuous variables, whereas categorical variables are presented as numbers and percentages. Comparisons of baseline characteristics according to groups were conducted by using Wilcoxon or Kruskal–Wallis tests for continuous variables and the Fisher exact test or χ^2^ test for categorical variables. Dunn post hoc test with Bonferroni correction were applied when required.

#### Primary outcomes

We used six methods to assess the primary objective by describing the different modes of interpretation of the lactate evolution measured over the first seven days on 30-day survival.

1. Lactate evolution over the first seven days according to 30-day status and intervention groups: A linear mixed model tested with a Kenward-Roger’s F test was used to analyze differences in lactate levels between survivors and non-survivors and according to hypothermia and9 normothermia intervention groups over the first seven days.
2. 30-day survival curves according to lactate level measured at baseline: baseline lactate was dichotomized to normolactatemia (≤ 2.2 mmol/L) and hyperlactatemia (> 2.2 mmol/L) and a Kaplan–Meier survival curve was drawn.
3. 30-day risk of death according to lactate level measured at any time over first seven days: To determine the association of lactate levels and the 30-day risk of death, a Cox model with lactate measured over the first seven days as a time-dependent variable was performed.
4. 30-day risk of death according to lactate level at baseline and late lactate re-increase: to assess the impact of a late increase in lactate level from baseline, a Cox model was applied. The dichotomized baseline level (normolactatemia and hyperlactatemia) was used as a time fixed variable and the first change from a normal to a hyperlactatemia level (whatever the lactate level at baseline) was used as a time-dependent variable. Corresponding survival curves were drawn.
5. 30-day risk of death according lactate trajectories over the first seven days: a latent class analysis was undertaken to identify three distinct lactate trajectories. Briefly, with this method, heterogeneity in lactate variation over the first seven days and identification of patients with similar trajectories were determined (See Additional file 1: Data method). Survival curves according to the identified lactate trajectories were drawn.
6. Joint model associating longitudinal lactate measurements over the first seven days and 30-day survival model.

Initially, a non-linear mixed model was applied to the lactate measurements over this period. This model included random intercepts for each subject. However, due to issues with non-convergence, it did not incorporate random slopes for time as a predictor variable within each subject. Subsequently, a 30-day survival analysis was conducted. The final step involved fitting the joint model, postulating a time-dependent relationship with a piecewise constant baseline risk function.

#### Adjustments on confounding factors

Adjusted analyses based on imputed data sets were performed with the following variables known to be associated with mortality or morbidity in CS and used in the HYPOECMO trial: age, prior myocardial infarction, prior cardiac arrest, post-cardiac surgery, vasopressor dose (representing the sum of epinephrine and norepinephrine doses at baseline; patients with no vasopressor therapy were coded as a dose of 0 µg/kg/min) and SOFA score at baseline. For all Cox models, Hazard ratio and 95% confidence interval were calculated from Cox models. Assumptions of hazards proportionality were checked.

#### Exploratory analysis

A time-dependent survival analysis investigating the association between serial lactate measurements from day 0 to day 7 and the onset of ischemic or hemorrhagic events was performed. The occurrence of death during the first seven days was considered a competing event, leading to the implementation of a time-dependent Fine and Gray model. When a patient presented two events, only the first one was considered.

#### Missing data management

For descriptive analysis, only patients with available baseline lactate were included. From the following next 7 days 3.4% of lactate values were missing (Additional file 1: Fig. S1). Therefore, no imputation was performed for the descriptive analysis. Conversely for the adjusted analyses on confounding variables, imputations on missing data were performed (Additional file 1: Data method). For all adjusted Cox analysis, we performed an adjusted model in each imputed dataset and pooled the results by applying Rubin’s rules.

A two-sided p-value ≤ 0.05 was regarded as statistically significant. Statistical analyses were performed using R, version 4.1.1 (2021-08-10) (R Foundation for Statistical Computing, Vienna, Austria).

## Results

### Population characteristics

The overall baseline characteristics of the studied population have previously been published [11]. Among the 334 patients included in the HYPOECMO trial, 318 had baseline lactate available of whom 160 were included in the moderate hypothermia group and 158 in the normothermia group (Table 1). Median age was 60 (IQR: 50–66) years and 25% were female gender. The most prevalent aetiology of CS was an acute coronary syndrome (114 patients, 36%). A total of 151 (47%) patients had a cardiac arrest prior to inclusion. Median LVEF before VA-ECMO was 20% (15–30). Norepinephrine and epinephrine treatments were required in 238 patients (77%) and 80 patients (26%) respectively. Regarding the initial study intervention, no difference was found in lactate course between moderate hypothermia and normothermia groups (Additional file 2: Fig. S2).

**Table 1:** Baseline characteristics according to baseline lactate status measured just before randomization

Variable	N	Total population	N	Hyperlactatamia	N	Normolactatemia	p-value
Delay Implantation-Randomization (min)	318	190 (104–278)	252	192 (100–272)	66	182 (105–286)	0.98
Normothermia group (%)	318	158 (50%)	252	124 (49%)	66	34 (52%)	0.74
Hypothermia group (%)	318	160 (50%)	252	128 (51%)	66	32 (48%)	
Demographics							
Age (years)	318	60 (50–66)	252	59 (49–66)	66	61 (57–68)	0.12
Female gender (%)	318	79 (25%)	252	62 (25%)	66	17 (26%)	0.85
BMI (Kg/m^2^)	308	26 (23–30)	243	26 (23–30)	65	25 (23–30)	0.51
Medical history (%)							
History of hypertension	311	115 (37%)	247	94 (38%)	64	21 (33%)	0.44
History heart failure	306	64 (21%)	242	44 (18%)	64	20 (31%)	0.022
History myocardial infarction	304	46 (15%)	240	33 (14%)	64	13 (20%)	0.19
History of cardiac ischemia	305	70 (23%)	240	48 (20%)	65	22 (34%)	0.019
History of valvular disease	305	38 (12%)	241	29 (12%)	64	9 (14%)	0.66
Causes (%)							
Cardiac arrest	318	151 (47%)	252	128 (51%)	66	23 (35%)	0.021
Acute coronary syndrome	318	114 (36%)	252	99 (39%)	66	15 (23%)	0.013
Valvular disease	318	27 (8%)	252	22 (9%)	66	5 (8%)	0.76
Toxic cardiomyopathy	318	8 (3%)	252	5 (2%)	66	3 (5%)	0.37
Ischemic cardiomyopathy	318	72 (23%)	252	54 (21%)	66	18 (27%)	0.31
Dilated cardiomyopathy	318	25 (8%)	252	20 (8%)	66	5 (8%)	0.92
Adrenergic and takotsubo cardiomyopathy	318	11 (3%)	252	10 (4%)	66	1 (2%)	0.47
Post cardiac surgery	318	47 (15%)	252	37 (15%)	66	10 (15%)	0.92
Rhymthmic cardiopathy	318	41 (13%)	252	30 (12%)	66	11 (17%)	0.30
Pulmonary embolism	318	17 (5%)	252	15 (6%)	66	2 (3%)	0.54
Myocarditis	318	30 (9%)	252	29 (12%)	66	1 (2%)	0.013
Other	318	110 (35%)	252	94 (37%)	66	16 (24%)	0.047
Baseline characteristics and management							
LVEF before VA-ECMO implantation (%)	201	20 (15–30)	162	20 (15–35)	39	20 (15–30)	0.46
Norepinephrine (%)	310	238 (77%)	244	184 (75%)	66	54 (82%)	0.27
Epinephrine (%)	310	80 (26%)	244	76 (31%)	66	4 (6%)	< 0.0001
Dobutamine (%)	310	192 (62%)	244	145 (59%)	66	47 (71%)	0.080
pH	317	7.32 (7.23–7.43)	251	7.30 (7.20–7.42)	66	7.38 (7.32–7.46)	< 0.0001
Lactate (mmol/L)	318	4.85 (2.60–8.20)	252	6.27 (3.85–9.35)	66	1.60 (1.40–2.00)	< 0.0001
Outcomes							
VA-ECMO duration (days)	276	6 (4–9)	216	6 (4–9)	60	6 (5–8)	0.40
In-ICU LOS (days)	318	12 (7–22)	252	12 (7–22)	66	14 (9–23)	0.080
Hospital LOS (days)	318	20 (9–46)	252	19 (8–43)	66	24 (11–49)	0.056
Renal replacement therapy (%)	313	130 (42%)	247	110 (45%)	66	20 (30%)	0.037
Mechanichal ventilation duration (days)	313	9 (5–17)	247	9 (5–17)	66	8 (4–19)	0.91
30-day non-survivor (%)	318	146 (46%)	252	127 (50%)	66	19 (29%)	0.002

Normolactatemia was defined as lactate level ≤ 2 mmol/L and hyperlactatemia was defined as lactate level > 2 mmol/L. Continuous variables are presented with median and IQR

### Lactate levels over time according to survival is described in Fig. 1

**Fig. 1:**
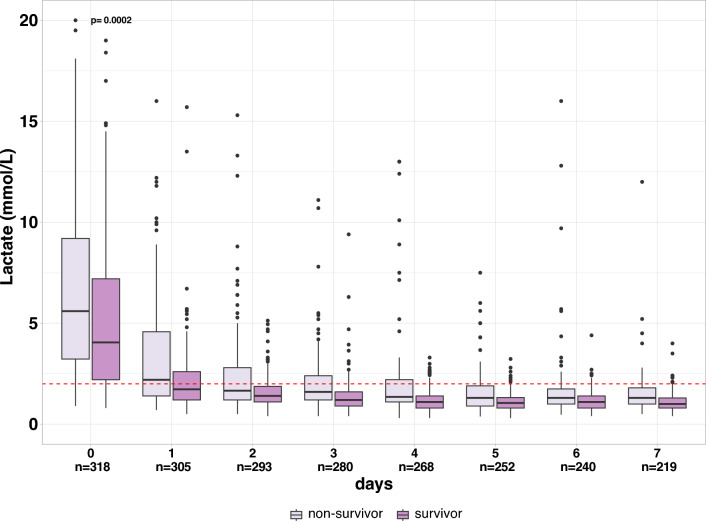
Lactate course from baseline to day seven according to 30-day status. One value out of range not represented. Red dashed line represents the 2 mmol/L threshold

At each day, non-survivors had higher lactate levels (p interaction = 0.0002).

### Baseline characteristics according to lactate at baseline dichotomized as normolactatemia (≤ 2 mmol/L) and hyperlactatemia (> 2 mmol/L)

**Fig. 2:**
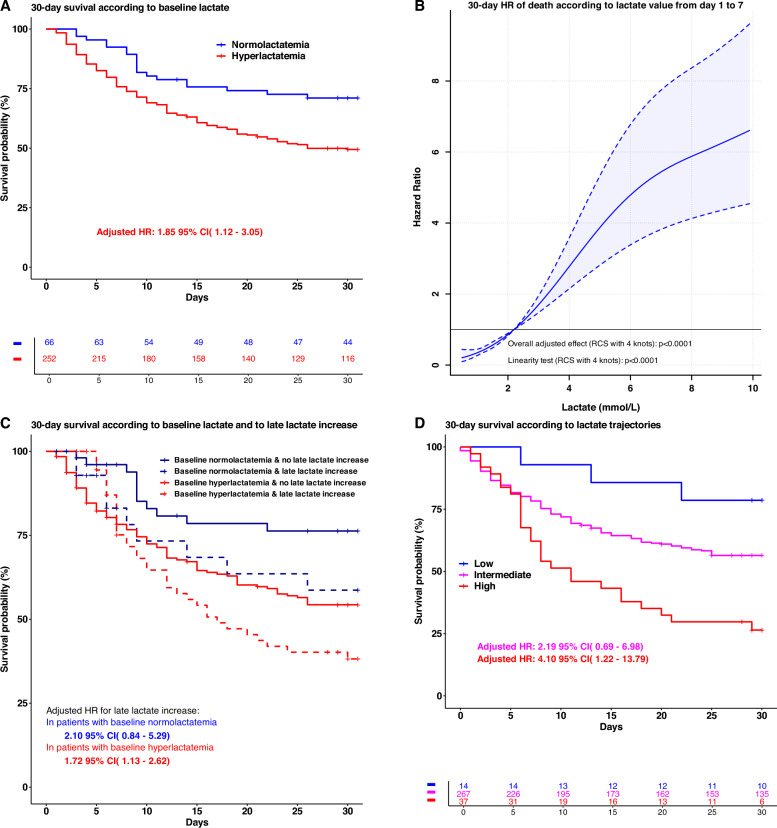
**A** Kaplan–Meier analysis for 30-day all-cause mortality according to hyperlactatemia status (> 2.2 mmol/L) versus normolactatemia (≤ 2.2 mmol/L) at baseline. **B** Hazard ratio of 30-day mortality according to lactate value measured at any time over the first seven days (time-dependent analysis). **C** Cox model analysis according to lactate at baseline (time-fixed variable) and to late lactate re-increase over the first 7 days (time dependent variable). **D**: 30-day mortality according to three trajectories of lactate evolution over the first seven days according to latent class analysis method. HR, hazard ratio. Adjusted p values were calculated from imputed data in all panels

**Fig. 3:**
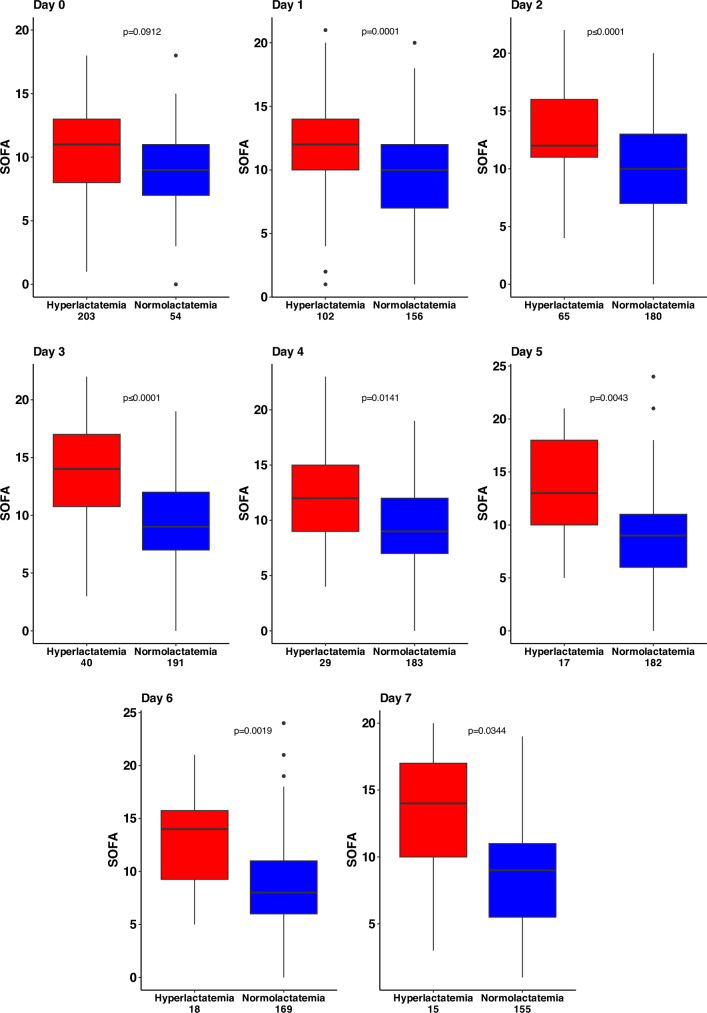
Daily SOFA scores according to normal versus abnormal lactate levels. p values were adjusted for multiplicity

At baseline, 66 patients had a normal lactate level (21%) (Table 1). There was no difference in the delay ECMO start to randomization between the two groups (p = 0.98). Briefly, patients with baseline hyperlactatemia had more comorbidities and more frequent a history of heart failure (p = 0.022) and cardiac ischemia (p = 0.019). Also, these patients more frequently presented with an acute coronary syndrome (p = 0.013) and cardiac arrest (p = 0.021). In addition, these patients more frequently required epinephrine treatment prior to the initiation of VA-ECMO. (p < 0.0001). However, after the first day, the administration of epinephrine did not significantly impact the lactate levels, which remained below 2 mmol/L in the subsequent days. (Additional file 1: Fig. S3). Importantly, 30-day mortality was higher (50% versus 29%, p = 0.002) for patients with baseline hyperlactatemia. The Kaplan–Meier curve for 30-day all-cause shows that hyperlactatemia was associated with an increased hazard ratio of death (adjusted HR: 1.85 95% CI (1.12–3.05), p = 0.016) (Fig. 2A). Patients with baseline hyperlactatemia had significantly higher SOFA scores over the first seven days (Fig. 3).

### Prognostic value of changes in lactate levels

Additional file 1: Table S1 depicts the lactate changes during the study according to the baseline lactate status. The decrease in lactate levels were always higher in patients with hyperlactatemia than in those with normolactatemia at baseline (p < 0.001). The decrease in lactate level at day 1 was not statistically different according to 30-day status (survivors: 56% (22–74) versus non-survivors 51% (15–72), p = 0.56). In patients with baseline hyperlactatemia, the change in lactate from baseline to day 1 was significantly higher in 30-day survivors (63% (48–77)) compared to 30-day non-survivors (57% (21–76), p = 0.027).

### Prognostic value of lactate levels

Figure 2B shows the association between lactate level at any time over the first seven days and the 30-day risk of death.

### Impact of lactate at baseline and late lactate increase

Figure 2C, describes four groups according to the baseline lactate status and the first lactate-increase thereafter (*i.e.,* a late lactate increases preceded by at least one normal lactate value). Moreover, patients who experienced a late lactate re-increase (n = 75, either the patients were with normal baseline normolactatemia or with baseline hyperlactatemia who normalized) over the seven first days had worst prognosis (adjusted HR: 1.78 95% CI (1.21–2.61), p = 0.004) regardless of the baseline lactate level status (p for interaction = 0.78).

### Impact of lactate trajectories on 30-day survival

Finally, we used a time latent class analysis, a method allowing to individualize trajectories regardless of the patient’s outcome, to characterize lactate trajectories over the first seven days. Three trajectories were identified, as depicted in Additional file 1: Fig. S4. While median lactate at baseline in each trajectory was increased (low: 5.1 (2.2–7) mmol/L versus intermediate: 4.7 (2.5–7.9) mmol/l versus high: 6.9 (3.9–10.9) mmol/L; p = 0.017), trajectory 1 defined patients remaining at high lactate values (n = 37), trajectory 2 defined patients remaining at intermediate lactate levels (n = 267) and trajectory 3 defined patients returning quickly to normal lactate levels (n = 14). Baseline characteristics according to these three trajectories were presented in Additional file 1: Table S2. No survival difference was found between low and intermediate lactate trajectory (adjusted HR at 2.19 (0.69–6.98), p = 0.18). When compared to low lactate trajectory, high lactate trajectory had a lower 30-day survival with an adjusted HR at 4.10 (1.22–13.79), p = 0.023) (Fig. 2D).

### Association between longitudinal lactate measurement and 30-day survival

Additional file 2: Fig. S5 and Additional file 3: Fig. S6, representing two actual patients within the cohort, illustrate how lactate patterns influence the 30-day survival risk. Additional file 2: Fig. S5 depicts a typical pattern of a low lactate trajectory, which is consistently associated with high survival rates at each lactate measurement timepoint. Conversely, Additional file 3: Fig. S6 presents a typical pattern of a high lactate trajectory, which, at each measurement timepoint, is associated with lower survival rates.

### Association between longitudinal lactate measurements and seven-day occurrence of ischemic or hemorrhagic events

During the first seven days, there were 17 cases of hemorrhagic shock, 17 cases of limb ischemia, and 6 cases of intestinal ischemia, totaling 40 ischemic or hemorrhagic events. Over the period, 73 deaths occurred of which 16 were due to a hemorrhagic or ischemic event. A trend towards an association was observed between lactate measurements over the first seven days and the occurrence of ischemic or hemorrhagic events within this timeframe, with a sub-distributional HR of 1.06 (95% CI [0.99–1.12]), and a p-value of 0.0966.

## Discussion

The present study, one of the largest in CS patients requiring VA-ECMO, shows that (i) no difference was found in lactate course between moderate hypothermia and normothermia groups and (ii) that the initial lactate level and the changes over time are associated with 30-day outcome parameters. The study findings provide some novel insights, regarding the trend profile and the relevance of a second peak during the 7 day period after ECMO start. Patients that initially normalized their lactate level or had a normal level at baseline but then had a subsequent increase in lactate to an abnormal level had a particularly worst prognosis.

### Lactate and hypothermia

While there is speculation that Targeted Temperature Management (TTM) may reduce metabolism, its impact on human lactate metabolism is complex and not fully understood. A retrospective analysis of the TTM study [19] revealed that TTM at 33 °C was associated with increased lactate levels during both the cooling and rewarming phases. Interestingly, these heightened lactate levels persisted for several days following the cessation of TTM [20]. This phenomenon could be elucidated, as elegantly shown by Beske et al*.* [21], by an upregulation of aerobic glycolysis pathways during hypothermia. In our study, the lactate trajectory appeared similar between the two groups. However, it's worth noting that our study was not specifically designed to explore the impact of temperature on alterations in lactate and in glucose metabolisms.

### Lactate evolution during VA-ECMO

The initial lactate value and its subsequent changes in VA-ECMO patients likely differ from those in CS not supported by VA-ECMO. Several reasons account for this: First, VA-ECMO patients typically exhibit a more pronounced myocardial dysfunction, often accompanied by extremely low cardiac output. This is coupled with significant kidney and liver injuries, both organs crucial for lactate metabolism. Second, for many of these patients, VA-ECMO is initiated during cardiopulmonary resuscitation. This scenario is frequently linked with increased ischemia–reperfusion injuries, higher epinephrine administration, and elevated lactate levels. Finally, worsening CS necessitating VA-ECMO support often demands higher catecholamine doses, which correlate with augmented lactate production [22]. The metabolism of lactate during VA-ECMO might be influenced by various factors. These include the efficiency of VA-ECMO (manifested by catecholamine reduction and restoration of blood flow), the severity of liver failure, and the emergence of complications such as septic or hemorrhagic shock, leg ischemia, and mesenteric ischemia. Consequently, a patient might initially experience a decline in lactate levels due to the restoration of systemic blood flow, followed by a subsequent rise owing to complications or organ failure. Finally, regarding the initial study intervention, no difference was found in lactate course between moderate hypothermia and normothermia groups.

### Previous studies and lactate in CS patients supported by VA-ECMO

While, globally, lactate measurements can provide prognostic insights for individuals experiencing CS, it is still uncertain whether peak serum lactate, lactate clearance, or measurements obtained at specific time points after VA-ECMO implantation offer the most accurate prognostic value. Existing data on the prognostic value of lactate and its decrease during CS with VA-ECMO support are limited, often stemming from small, monocentric, and retrospective studies. The recent multicenter study assessing the effect of VA-ECMO on mortality in infarct-related CS found high baseline lactate levels. However, 77% of their cohort consisted of post cardiac arrest CS patients whereas, in our study, only 45% had experienced post cardiac arrest CS [23]. Both the ENCOURAGE score [14] and the PREDICT score [15] utilize pre-ECMO lactate values. Initial lactate levels and their early evolution (12–24 h) have been linked to prognosis [6, 24]. However, findings regarding lactate changes over time remain inconclusive. Two studies propose that a delayed measurement (72 h) might offer insights into the 30-day prognosis. Finally, using a novel artificial intelligence-driven tool for predicting in-hospital mortality of patients receiving VA-ECMO, the ECMOPAL study found that the most predictive variable was lactate [25].

### Lactate decrease and 30-day mortality

A recent meta-analysis and systematic review have brought to light that a reduction in lactate levels is associated with improved prognoses in CS, which includes a subgroup of patients supported by VA-ECMO [16]. However, the studies included in the meta-analysis exhibited substantial heterogeneity in terms of treatment approaches and mortality endpoints. Notably, most of these studies were small-scale, single-center, and retrospective in nature. Furthermore, data specifically focusing on patients receiving VA-ECMO support showed inconsistencies. While our study's findings on day 1 lactate clearance align with prior observations in CS (60%) [16], we did not identify any significant differences between survivors and non-survivors. Nevertheless, we consistently observed higher lactate reductions from day one to day seven in patients with baseline hyperlactatemia. Specifically, among those with initial hyperlactatemia, a reduction in lactate levels on day one was positively associated with 30-day mortality.

### Significance of initial lactate levels

Initial lactate levels have proven to be of substantial importance in various patient cohorts, encompassing those with septic, cardiogenic, and hemorrhagic shock, as they serve as predictive markers for outcomes. However, it's noteworthy that severe CS can sometimes manifest without elevated lactate levels. In a post-hoc analysis [2] of the DOREMI trial [26], it was revealed that 26% of patients presented with baseline normolactatemia, which was associated with a 30-day hospital mortality rate of 32%, in contrast to the 42% mortality rate in hyperlactatemic patients. Interestingly, 21% of our VA-ECMO patients, despite having high SOFA scores, exhibited baseline lactate levels below 2.2 mmol/L. This observation is unexpected for critically ill patients of this nature. One plausible explanation may be the time lapse between ECMO initiation and the initial lactate measurement, which might have led to a slight normalization of initially elevated lactate levels in some patients (192 min versus 180 min). It's crucial to note that this delay was consistent across both patient groups. Reflecting the findings of the post-hoc DOREMI trial analysis [2], patients with normal lactate levels experienced more favorable outcomes (30-day mortality: 29% versus 50%, p = 0.002).

### Lactate dynamics and its importance

We employed a time-dependent model to comprehend the temporal evolution of lactate and its connection to mortality. The presence of hyperlactatemia at any point during the initial seven days emerged as a predictor of unfavorable outcomes, implying that not only the initial measurement but also subsequent increases or sustained hyperlactatemia hold prognostic significance. By distinguishing between the initial lactate values and subsequent rises, we were able to identify four distinct lactate kinetic patterns. Notably, a secondary increase was linked to poorer outcomes, regardless of the initial lactate value. These findings were further supported by lactate trajectories analysis and joint model analysis, where an elevated trajectory was associated with the most adverse outcomes.

### Lactate as a danger biomarker of ischemic or hemorrhagic events

We found a trend association between serial lactate measurements and the onset of ischemic or thrombotic events during the first seven days. These events occur very frequently in patients on VA-ECMO and are associated with prognosis. In a cohort of 11,984 adults on VA-ECMO, 8457 ischemic or hemorrhagic events occurred, 62% were hemorrhagic events [27]. Although, in this study, we didn’t reach the statistical significance, considering the potential implications for patient management, this result suggests that lactate could be considered a marker of risk for these two events.

### Strengths and study limitations

On the one hand, the HYPO-ECMO study was designed as a multicenter, randomized, prospective trial, which effectively minimized bias. On the other hand, our study, being a post-hoc analysis, carries significant limitations compared to a pre-planned analysis. These include the risk of selection and confirmation bias, an increased risk of type I errors, an inability to control for confounding variables, and a lack of external validation. All these factors limit the generalizability of a post-hoc analysis. One of the notable strengths of our study was the comprehensive monitoring of lactate levels over a seven-day period in nearly all participants of the HYPO-ECMO trial. However, we acknowledge a limitation due to the inclusion of patients with various underlying causes of CS, which introduced heterogeneity into the study population. Given the infrequent occurrence of CS necessitating VA-ECMO treatment, conducting a study focused on a specific subset within this population, apart from acute myocardial infarction, would be challenging. Another limitation was the protocol-mandated 24 h delay post-baseline for the initial lactate measurement, which may have missed early and significant lactate level variations. Nevertheless, despite these limitations, the current findings are biologically plausible and align with the well-described lactate kinetics described in non-assisted CS.

## Conclusions

In our post-hoc analysis of the HYPO-ECMO study, we established a connection between the temporal changes in lactate levels and various outcome parameters. Particularly, we found that a late increase in lactate was linked to a less favorable prognosis. These findings underscore the significance of monitoring lactate kinetics as valuable prognostic markers in cases of CS requiring VA-ECMO support. They also suggest the potential for lactate kinetics to serve as targets for future therapeutic research in this patient population.

### Supplementary Information

**Additional file 1: Figure S1.** Lactate missing data from day one to day seven among the 318 patients with available baseline lactate values. **Figure S2.** Lactate course over time according to 30-day status and according to randomization groups. Red dashed line represents the 2 mmol/L threshold. One value out of range not represented. **Figure S3.** Evolution of lactate level according to epinephrine administration per day over the first seven days. p interaction was obtained from linear mixed model while each p value at bottom were obtained from adjusted Wilcox tests. n are presented under each day for each group and read dashed line represents the 2 mmol/L threshold. **Figure S4.** Lactate trajectories from baseline to day seven. One value out of range (40 mmol/l). **Table S1.** Lactate decrease from day one to day seven according to lactate status at baseline. **Table S2.** Baseline characteristics according to the three lactate trajectories identified with the time latent class analysis.

**Additional file 2: Figure S5.** Joint model for a typical pattern of a low lactate trajectory. The animated figure is provided in a separated file.

**Additional file 3: Figure S6.** Joint model for a typical pattern of a high lactate trajectory. The animated figure is provided in a separated file.

## Data Availability

On request to the first author (blevy5463@gmail.com).
