# Supplementary material for: Serial daily lactate levels association with 30-day outcome in cardiogenic shock patients treated with VA-ECMO: a post-hoc analysis of the HYPO-ECMO study
Source: Ann Intensive Care. 2024 Mar 27;14:43. doi: 10.1186/s13613-024-01266-6 (PMC10973308; doi:10.1186/s13613-024-01266-6)
Supplement: Supplementary file 1 — Additional file 1: Figure S1. Lactate missing data from day one to day seven among the 318 patients with available baseline lactate values. Figure S2. Lactate course over time according to 30-day status and according to randomization groups. Red dashed line represents the 2 mmol/L threshold. One value out of range not represented. Figure S3. Evolution of lactate level according to epinephrine administration per day over the first seven days. p interaction was obtained from linear mixed model while each p value at bottom were obtained from adjusted Wilcox tests. n are presented under each day for each group and read dashed line represents the 2 mmol/L threshold. Figure S4. Lactate trajectories from baseline to day seven. One value out of range (40 mmol/l). Table S1. Lactate decrease from day one to day seven according to lactate status at baseline. Table S2. Baseline characteristics according to the three lactate trajectories identified with the time latent class analysis. [file 13613_2024_1266_MOESM1_ESM.docx]

**Additional file 1: Data material**

**Serial daily lactate levels association with 30-day outcome in cardiogenic shock patients treated with VA-ECMO: A post-hoc analysis of the HYPO-ECMO study**

Summary

[Statistical method 3](#_Toc153535306)

[Time latent class analysis 3](#_Toc153535307)

[Imputation method 3](#_Toc153535308)

[Additional file Figures 5](#_Toc153535309)

[Figure S1: Lactate missing data from day one to day seven among the 318 patients with available baseline lactate values. 5](#_Toc153535310)

[Figure S2: Lactate course over time according to 30-day status and according to randomization groups. Red dashed line represents the 2mmol/L threshold. One value out of range not represented. 6](#_Toc153535311)

[Figure S3: Evolution of lactate level according to epinephrine administration per day over the first seven days. p interaction was obtained from linear mixed model while each p value at bottom were obtained from adjusted Wilcox tests. n are presented under each day for each group and read dashed line represents the 2 mmol/L threshold. 7](#_Toc153535312)

[Figure S4: Lactate trajectories from baseline to day seven. One value out of range (40 mmol/l) 8](#_Toc153535313)

[Figure S5: Joint model for a typical pattern of a low lactate trajectory. The animated figure is provided in a separated file (Additional file_figure_S5.gif) 9](#_Toc153535314)

[Figure S6: Joint model for a typical pattern of a high lactate trajectory. The animated figure is provided in a separated file (Additional file_figure_S6.gif) 9](#_Toc153535315)

[Additional file Tables 10](#_Toc153535316)

[Table S1: Lactate decrease from day one to day seven according to lactate status at baseline 10](#_Toc153535317)

[Table S2: Baseline characteristics according to the three lactate trajectories identified with the time latent class analysis. 11](#_Toc153535318)

# Statistical method

## Time latent class analysis

A latent class analysis was undertaken to identify lactate trajectories over time. Briefly, with this method, heterogeneity in lactate variation over time and identification of patients with similar trajectories were determined. In other words, patients with similar lactate levels over time were classified in the same trajectory.

We first identified which link functions (linear link function, non-linear link function such as Beta cumulative distribution function or Quadratic I-splines) best fit the data. Then, to select the most appropriate link function, models were compared in terms of goodness-of-fit using measures such as AIC SABIC or BIC. Models were compared with increasing numbers of groups. To select the most appropriate number of groups, different models were compared according to entropy criterion (the probability that a individual is assigned to the right class) and of goodness-of-fit using criteria. The goal is to find the model with the best trade-off between low of goodness-of-fit criteria, high entropy and reasonable percentage of patients per class (eg: a class with < 5 patients might not be meaningful or useful).

## Imputation method

For adjusted analyses on confounding variables, imputations on missing data were performed. Among the variables used as adjustment variables, four variables had missing data: prior MI, vasopressor dose, lactate and SOFA score (calculated using six components). To preserve the completeness of the data for the adjusted analyses, we used Multiple Imputation by Chained Equations (MICE) to impute missing values. We performed multiple imputation with the R package mice by generating 100 imputed datasets with 50 iterations. The following variables were used as predictors: age, gender, BMI, history of CV disease (hypertension, HF, MI, ischemic cardiopathy, valvular cardiopathy), prior cardiac arrest, CS etiologies (acute coronary syndrome, ischemic cardiomyopathy, post cardiac surgery, rhythmic cardiopathy, other etiologies), vasopressor dose, dobutamine dose, pH, lactate and the six components of SOFA score. No outcome data were included. No auxiliary variables and interaction terms were applied. Imputation was performed by predictive mean matching for quantitative variables and by logistic regression for binary variables. After imputation, we calculated SOFA score in each imputed dataset.

# **Additional file** Figures

## Figure S1: Lactate missing data from day one to day seven among the 318 patients with available baseline lactate values.


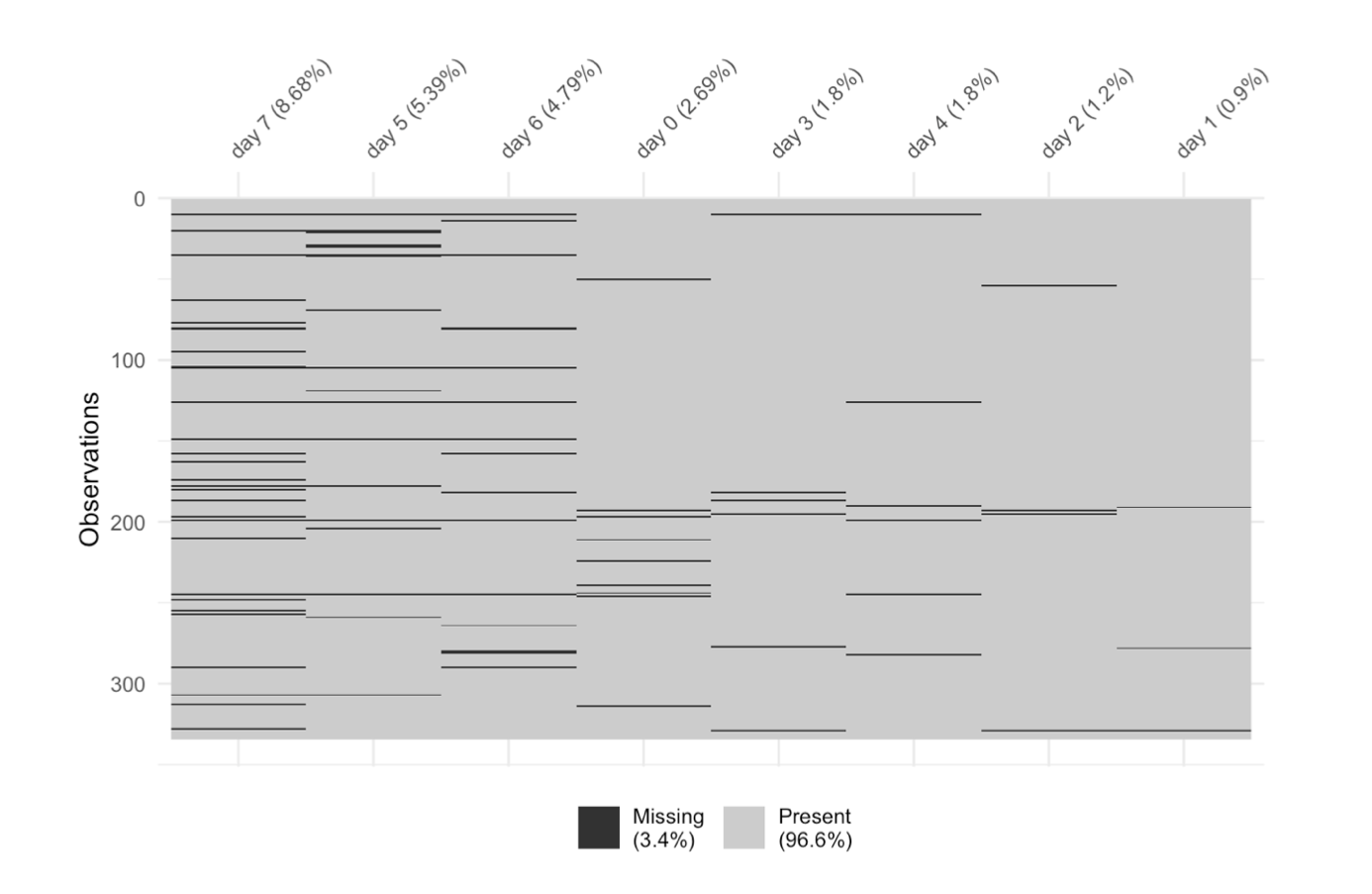


## Figure S2: Lactate course over time according to 30-day status and according to randomization groups. Red dashed line represents the 2mmol/L threshold. One value out of range not represented.

## Figure S3: Evolution of lactate level according to epinephrine administration per day over the first seven days. p interaction was obtained from linear mixed model while each p value at bottom were obtained from adjusted Wilcox tests. n are presented under each day for each group and read dashed line represents the 2 mmol/L threshold.

## Figure S4: Lactate trajectories from baseline to day seven. One value out of range (40 mmol/l)


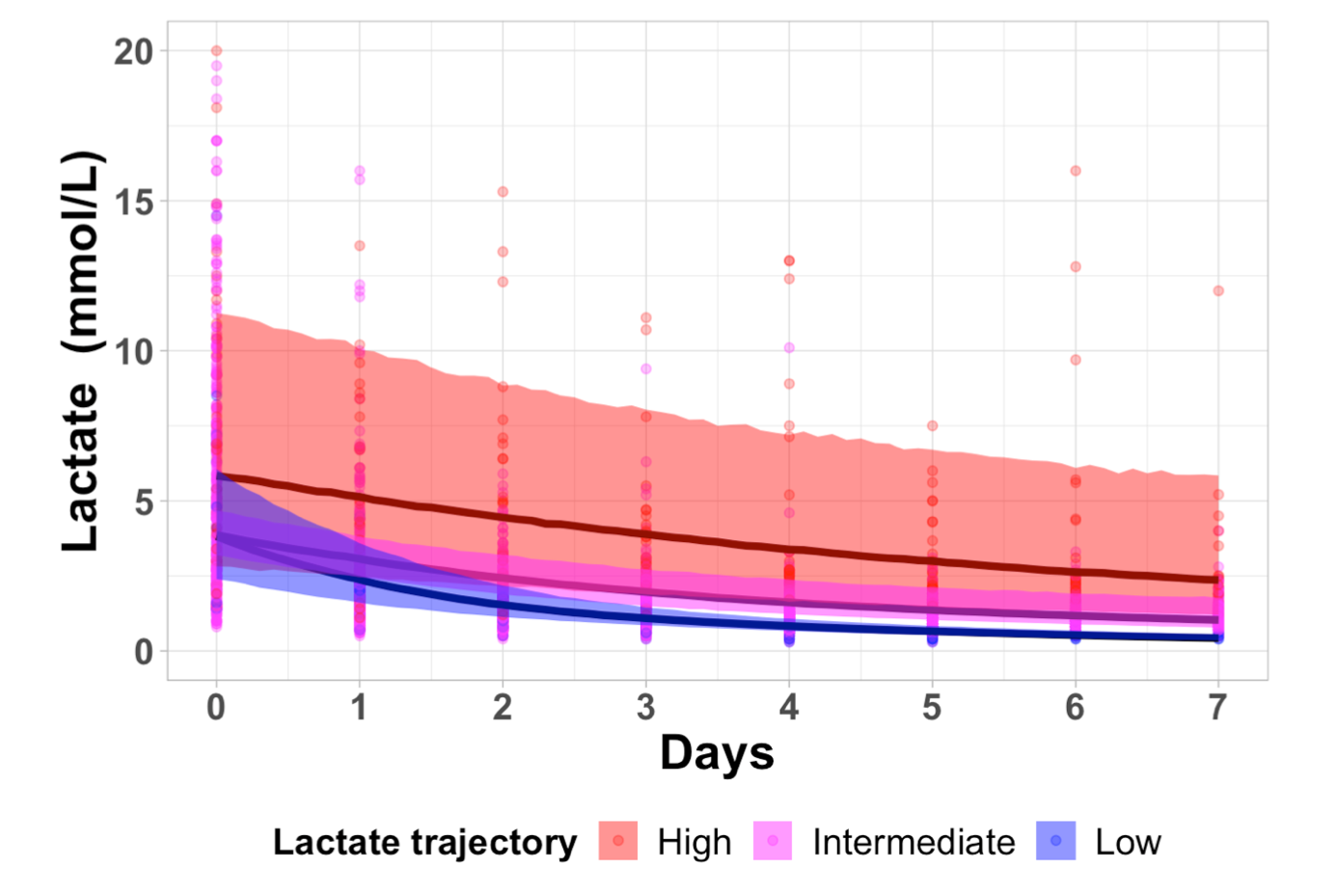


## Figure S5: Joint model for a typical pattern of a low lactate trajectory. The animated figure is provided in a separated file (**Additional file**_figure_S5.gif)

## Figure S6: Joint model for a typical pattern of a high lactate trajectory. The animated figure is provided in a separated file (**Additional file**_figure_S6.gif)

# **Additional file** Tables

## Table S1: Lactate decrease from day one to day seven according to lactate status at baseline

| **Variable in %** | **N** | **Total population** | **N** | **Hyperlactatamia** | **N** | **Normolactatemia** | **p-value** |
| --- | --- | --- | --- | --- | --- | --- | --- |
| Lactate decrease at day 1 | 305 | 54 (18 - 73) | 239 | 62 (37 - 76) | 66 | 14 (-11 - 36) | <0.0001 |
| Lactate decrease at day 2 | 291 | 64 (37 - 80) | 227 | 71 (57 - 83) | 64 | 20 (0 - 44) | <0.0001 |
| Lactate decrease at day 3 | 278 | 69 (45 - 81) | 216 | 76 (60 - 83) | 62 | 33 (0 - 50) | <0.0001 |
| Lactate decrease at day 4 | 265 | 71 (50 - 85) | 202 | 80 (64 - 87) | 63 | 39 (11 - 55) | <0.0001 |
| Lactate decrease at day 5 | 249 | 74 (50 - 86) | 188 | 81 (67 - 87) | 61 | 39 (13 - 56) | <0.0001 |
| Lactate decrease at day 6 | 237 | 74 (50 - 85) | 179 | 80 (67 - 87) | 58 | 43 (13 - 56) | <0.0001 |
| Lactate decrease at day 7 | 216 | 74 (51 - 85) | 164 | 81 (67 - 87) | 52 | 37 (0 - 52) | <0.0001 |

## Table S2: Baseline characteristics according to the three lactate trajectories identified with the time latent class analysis.

| **variable** | **N** | **Total** | **N** | **High** | **N** | **Intermediate** | **N** | **Low** | **p-value** |
| --- | --- | --- | --- | --- | --- | --- | --- | --- | --- |
| Delay randomization-implantation (min) | 318 | 190 (104 - 278) | 37 | 218 (134 - 290) | 267 | 185 (100 - 278) | 14 | 152 (107 - 224) | 0.43 |
| Nomorthermia group | 318 | 158 (50 %) | 37 | 23 (62 %) | 267 | 127 (48 %) | 14 | 8 (57 %) | 0.21 |
| Hypothermia group | 318 | 160 (50 %) | 37 | 14 (38 %) | 267 | 140 (52 %) | 14 | 6 (43 %) |  |
| **Demographics** |  |  |  |  |  |  |  |  |  |
| Age (years) | 318 | 60 (50 - 66) | 37 | 59 (51 - 65) | 267 | 60 (50 - 66) | 14 | 56 (46 - 63) | 0.49 |
| Female gender (%) | 318 | 79 (25 %) | 37 | 11 (30 %) | 267 | 61 (23 %) | 14 | 7 (50 %) | 0.063 |
| Body mass index (Kg/m²) | 308 | 26 (23 - 30) | 35 | 24 (23 - 28) | 260 | 26 (23 - 30) | 13 | 26 (24 - 27) | 0.29 |
| **Medical history** |  |  |  |  |  |  |  |  |  |
| History of Hypertension | 311 | 115 (37 %) | 37 | 9 (24 %) | 260 | 103 (40 %) | 14 | 3 (21 %) | 0.092 |
| History of heart failure | 306 | 64 (21 %) | 37 | 8 (22 %) | 255 | 54 (21 %) | 14 | 2 (14 %) | 0.91 |
| History of myocardial infarction | 304 | 46 (15 %) | 36 | 4 (11 %) | 254 | 40 (16 %) | 14 | 2 (14 %) | 0.89 |
| History of cardiac ischemia | 305 | 70 (23 %) | 37 | 8 (22 %) | 254 | 58 (23 %) | 14 | 4 (29 %) | 0.88 |
| History of valvular disease | 305 | 38 (12 %) | 37 | 9 (24 %) | 254 | 27 (11 %) | 14 | 2 (14 %) | 0.057 |
| **Causes** |  |  |  |  |  |  |  |  |  |
| Cardiac arrest | 318 | 151 (47 %) | 37 | 16 (43 %) | 267 | 128 (48 %) | 14 | 7 (50 %) | 0.85 |
| Acute coronary syndrome | 318 | 114 (36 %) | 37 | 9 (24 %) | 267 | 102 (38 %) | 14 | 3 (21 %) | 0.13 |
| Valvular disease | 318 | 27 (8 %) | 37 | 6 (16 %) | 267 | 19 (7 %) | 14 | 2 (14 %) | 0.088 |
| Toxic cardiomyopathy | 318 | 8 (3 %) | 37 | 1 (3 %) | 267 | 6 (2 %) | 14 | 1 (7 %) | 0.24 |
| Ischemic cardiomyopathy | 318 | 72 (23 %) | 37 | 5 (14 %) | 267 | 65 (24 %) | 14 | 2 (14 %) | 0.27 |
| Dilated cardiomyopathy | 318 | 25 (8 %) | 37 | 5 (14 %) | 267 | 18 (7 %) | 14 | 2 (14 %) | 0.19 |
| Adrenergic and takotsubo cardiomyopathy | 318 | 11 (3 %) | 37 | 2 (5 %) | 267 | 8 (3 %) | 14 | 1 (7 %) | 0.27 |
| Post cardiac surgery | 318 | 47 (15 %) | 37 | 3 (8 %) | 267 | 42 (16 %) | 14 | 2 (14 %) | 0.51 |
| Rhymthmic cardiopathy | 318 | 41 (13 %) | 37 | 5 (14 %) | 267 | 34 (13 %) | 14 | 2 (14 %) | 0.82 |
| Pulmonary embolism | 318 | 17 (5 %) | 37 | 2 (5 %) | 267 | 15 (6 %) | 14 | 0 (0 %) | 1.00 |
| Myocarditis | 318 | 30 (9 %) | 37 | 9 (24 %) | 267 | 21 (8 %) | 14 | 0 (0 %) | 0.007 |
| Other | 318 | 110 (35 %) | 37 | 24 (65 %) | 267 | 80 (30 %) | 14 | 6 (43 %) | 0.0001 |
| **Baseline characteristics and management** |  |  |  |  |  |  |  |  |  |
| LVEF before VA-ECMO implantation (%) | 201 | 20 (15 - 30) | 27 | 18 (11 - 30) | 164 | 20 (15 - 30) | 10 | 25 (15 - 40) | 0.25 |
| Norepinephrine (%) | 310 | 238 (77 %) | 36 | 31 (86 %) | 260 | 197 (76 %) | 14 | 10 (71 %) | 0.33 |
| Epinephrine (%) | 310 | 80 (26 %) | 36 | 10 (28 %) | 260 | 65 (25 %) | 14 | 5 (36 %) | 0.58 |
| Dobutamine (%) | 310 | 192 (62 %) | 36 | 26 (72 %) | 260 | 157 (60 %) | 14 | 9 (64 %) | 0.38 |
| pH | 317 | 7.32 (7.23 - 7.43) | 36 | 7.26 (7.16 - 7.34) | 267 | 7.33 (7.23 - 7.43) | 14 | 7.37 (7.31 - 7.45) | 0.030 |
| Lactate (mmol/L) | 318 | 4.85 (2.60 - 8.20) | 37 | 6.90 (3.90 - 10.90) | 267 | 4.70 (2.50 - 7.90) | 14 | 5.10 (2.20 - 7.00) | 0.017 |
| **Outcomes** |  |  |  |  |  |  |  |  |  |
| VA-ECMO duration | 276 | 6 (4 - 9) | 34 | 8 (6 - 10) | 228 | 6 (4 - 9) | 14 | 5 (3 - 7) | 0.010 |
| In-ICU LOS (days) | 318 | 12 (7 - 22) | 37 | 9 (7 - 17) | 267 | 12 (8 - 24) | 14 | 12 (7 - 18) | 0.19 |
| Hospital LOS (days) | 318 | 20 (9 - 46) | 37 | 10 (7 - 22) | 267 | 22 (9 - 48) | 14 | 24 (14 - 42) | 0.038 |
| Renal replacement therapy (%) | 313 | 130 (42 %) | 37 | 25 (68 %) | 262 | 100 (38 %) | 14 | 5 (36 %) | 0.003 |
| Mechanichal ventilation duration (days) | 313 | 9 (5 - 17) | 37 | 9 (6 - 17) | 262 | 10 (5 - 17) | 14 | 6 (2 - 14) | 0.23 |
| 30-day non survivor (%) | 318 | 146 (46 %) | 37 | 27 (73 %) | 267 | 116 (43 %) | 14 | 3 (21 %) | 0.0006 |
