# Supplementary figures and images for: Serial daily lactate levels association with 30-day outcome in cardiogenic shock patients treated with VA-ECMO: a post-hoc analysis of the HYPO-ECMO study
Source: Ann Intensive Care. 2024 Mar 27;14:43. doi: 10.1186/s13613-024-01266-6 (PMC10973308; doi:10.1186/s13613-024-01266-6)

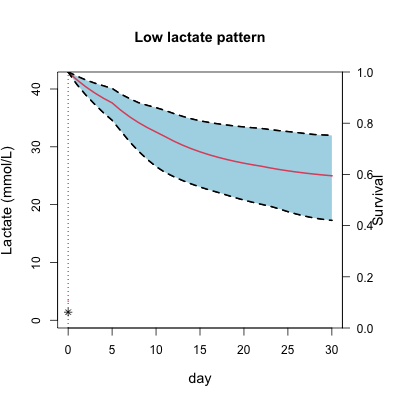

Supplement: Supplementary file 2 — Additional file 2: Figure S5. Joint model for a typical pattern of a low lactate trajectory. The animated figure is provided in a separated file. [file 13613_2024_1266_MOESM2_ESM.gif]

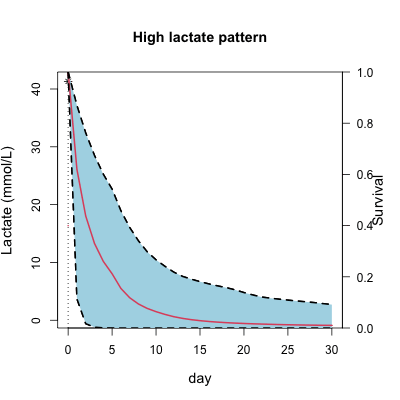

Supplement: Supplementary file 3 — Additional file 3: Figure S6. Joint model for a typical pattern of a high lactate trajectory. The animated figure is provided in a separated file. [file 13613_2024_1266_MOESM3_ESM.gif]
